# Molecular evidence of *Sarcocystis nesbitti* in water samples of Tioman Island, Malaysia

**DOI:** 10.1186/s13071-016-1883-9

**Published:** 2016-11-23

**Authors:** Shahhaziq Shahari, Tengku Idzzan Nadzirah Tengku-Idris, Mun Yik Fong, Yee Ling Lau

**Affiliations:** Department of Parasitology, Faculty of Medicine, University of Malaya, 50603 Kuala Lumpur, Malaysia

**Keywords:** *Sarcocystis*, Tioman Island, Water sample, *Sarcocystis nesbitti*, Sporocysts, Surveillance study

## Abstract

**Background:**

*Sarcocystis* are intracellular protozoan parasites that are characterised by their ability to invade muscle tissue and form intramuscular sarcocysts. A muscular sarcocystosis outbreak was reported by travellers returning from Tioman Island in 2011 and 2012 where *Sarcocystis nesbitti* was identified as the main cause. The source of the *S. nesbitti* that was involved has remained elusive, although water is hypothesised to be the main cause of transmission. A surveillance study was therefore undertaken in the northern regions of Tioman Island to identify the source of *S. nesbitti* by screening rivers, water tanks, wells and seawater.

**Methods:**

Water samples were collected from rivers, water tanks, wells and seawater on Tioman Island over the course of April to October 2015. Water samples were indirectly screened for *Sarcocystis* species by obtaining sediment from respective water sources. PCR amplification of the 18S rRNA gene region was conducted to identify positive samples. Microscopy was used in an attempt to reappraise PCR results, but no sporocysts were detected in any of the samples.

**Results:**

A total of 157 water samples were obtained and 19 were positive for various *Sarcocystis* species. Through BLASTn and phylogenetic analysis, these species were found to be *S. singaporensis*, *S. nesbitti*, *Sarcocystis* sp. YLL-2013 and one unidentified *Sarcocystis* species.

**Conclusions:**

This is the first positive finding of *S. nesbitti* in water samples on Tioman Island, which was found in a water tank and in river water samples. This finding supports the hypothesis that water was a potential medium for the transmission of *S. nesbitti* during the outbreak. This will potentially identify areas in which preventive measures can be taken to prevent future outbreaks.

## Background

*Sarcocystis* (*sarco* = flesh; *cystis* = cyst-forming) are intracellular protozoan parasites characterised by their ability to invade muscle tissue, where they mature into sarcocysts. The life-cycle of *Sarcocystis* involves an obligate prey-predator relationship with a definitive host (the predator) and an intermediate host (the prey). Upon ingestion of sarcocyst-infected muscle tissue from the intermediate host, sexual stages develop within the small intestine of the definitive host, and then sporulated oocysts containing sporocysts are expelled in the faeces. Ingestion of sporocysts by an intermediate host, i.e. the faecal-oral route, enables the asexual stages of *Sarcocystis* to develop, resulting in intramuscular sarcocysts [1].

Humans are the definitive host for at least two known *Sarcocystis* species, *S. hominis* and *S. suihominis*, and ingestion of undercooked beef and pork, respectively, that contain sarcocysts leads to intestinal sarcocystosis, which can induce symptoms such as nausea, vomiting and enteritis. Most cases, however, are presented as mild or asymptomatic [2]. Conversely, only one *Sarcocystis* species is known to utilize humans as intermediate hosts. This species is *S. nesbitti* and it was acknowledged to be involved in the largest known acute muscular sarcocystosis (AMS) outbreak, which occurred in 2011 and 2012 on Tioman Island, Malaysia [3].

In 2011 and 2012, GeoSentinel and TropNet reported a total of 100 cases involving foreigners travelling to Tioman Island during the summer months, i.e. from July to August. All patients exhibited two distinct symptoms, relapsing fever and myalgia, whilst a few reported suffering from arthralgia, asthenia, headache, cough and diarrhoea [3]. Additionally, facial swelling was reported in a few of the affected individuals. Histological examination of muscle biopsy samples that were obtained from six patients, stained with hematoxylin and eosin (H&E), revealed sarcocysts within the muscle fibres. Observation of the sarcocyst wall via electron microscopy and molecular characterisation via amplification of the 18S rRNA gene region revealed that *S. nesbitti* was the cause of the AMS outbreak [4].

*Sarcocystis nesbitti* utilises a snake-primate life-cycle [5–7], where humans are considered an aberrant host presenting intermediate host-like symptoms. As transmission of muscular sarcocystosis occurs via the faecal-oral route, infection probably occurred via ingestion of either food or water contaminated with *S. nesbitti* sarcocysts. Most water supplied to residents of Tioman Island comes from untreated environmental sources, i.e. gravity feed systems and tube wells [8]. It was therefore hypothesised that water contamination was the most probable cause of the AMS outbreak. A surveillance study conducted in November 2011, however, reported that all water samples tested negative for presence of *S. nesbitti* [8]. This was attributed to the water samples being too dilute, making it impossible to detect sporocysts via microscopy.

To overcome the diluting factor, this study screened water samples indirectly by obtaining sediment from respective water sources. As sporocysts are purified using floatation techniques [1], it was assumed that sporocysts in water would sink and be trapped in the sediment of water tanks and rivers with relatively stagnant water. In addition to microscopy, samples were screened via PCR due to its greater sensitivity when compared to microscopy alone.

In previous studies, the 18S rRNA gene has proven to be a suitable candidate for species identification [5, 9]. In this study, therefore, amplification of the 18S rRNA gene with subsequent BLASTn and phylogenetic analyses was conducted in order to identify potential *S. nesbitti* sources, and to also determine what other *Sarcocystis* species are present on the island.

## Methods

### Sample collection

Water samples were collected from rivers, water tanks, wells and beaches located in the northern villages of Tioman, Pahang, Malaysia from April to October 2015. These villages were Tekek Village, Salang Village, Juara Village and Ayer Batang Village as these villages were reported to have been highly frequented by the case patients during the muscular sarcocystosis outbreak [4]. Water samples were indirectly screened by obtaining sediment samples from respective water source and placed in 50 ml Falcon tubes. A total of 157 sediment samples were obtained. This included 127 river sediment samples, 25 water tank and well samples and 5 sediment samples obtained from seawater. As a previous surveillance study has noted that sporocyst concentration in water samples are very low [8], no preservatives were added to the samples as to not dilute the sample further. Samples were stored at 4 °C until analysed.

### DNA extraction

From the sediment, 0.25 g was used for DNA extraction for each sample. DNA extraction was performed using the PowerSoil® DNA Isolation Kit (MO Bio Laboratories, Carlsbad, USA) according to the manufacturer’s protocol. Samples were eluted at 100 μl and subsequently, DNA samples were then store at -20 °C.

### PCR amplification

Nested PCR was carried out using primers 1L (5′-CCA TGC ATG TCT AAG TAT AAG C-3′) and 1H (5′-TAT CCC CAT CAC GAT GCA TAC-3′) for the primary reaction and 3L (5′-CTA GTG ATT GGA ATG ATG GG-3′) and 2H (5′-ACC TGT TAT TGC CTC AAA CTT C-3′) for the secondary reaction [9]. Only 4 μl of template DNA eluted form the PowerSoil® extraction was used for each reaction. PCR reagents were mixed to have the following final concentrations per reaction: 1× Go*Taq*® Flexi Buffer, 4 mM MgCl_2_, 0.2 μM of each primer, 0.2 μM dNTP and 1U Go*Taq*® Flexi DNA Polymerase (Promega, Madison, USA). Nested PCR was then carried using the following protocol: 95 °C for 2 min, followed by 35 cycles of 94 °C for 40 s, 50 °C for 30 s, 72 °C for 1.5 min, and final extension at 72 °C for 10 min. The final PCR products were then viewed under gel electrophoresis on a 1–1.3% agarose gel with an expected band size of ~900 kb.

### Cloning and sequencing

Positive PCR samples were PCR purified using either the QIAquick PCR Purification Kit or the QIAquick Gel Extraction Kit (QIAGEN, Hilden, Germany) following the manufacturer’s protocol. Samples were then ligated into pGEM®-T Vector Systems (Promega) and transformed into TOP10 Competent cells. Colonies were checked via colony PCR and plasmids were extracted using the QIAprep Spin Miniprep Kit (QIAGEN). Lastly, plasmids were sequenced via Sanger sequencing through a commercial sequencing company using M13 forward and reverse primers.

### BLASTn analysis

Sequencing results were trimmed to omit flanking M13 regions. Forward and reverse sequences were then aligned and the electropherogram was checked to resolve any discrepancies between the aligned sequences. Sequencing results was then checked using Basic Local Alignment Search Tool (BLASTn) for the identification of species.

### Phylogenetic analysis

To reappraise the BLASTn results, trimmed sequences were aligned with 18S rRNA gene sequences of other *Sarcocystis* sp. obtained from GenBank using ClustalW. A phylogenetic tree was constructed using the Neighbour-Joining method with a bootstrap value of 1000 using the MEGA 6.06 software. The 18S rRNA gene sequence of *Eimera tenella* was used as the outgroup.

## Results

A total of 157 sediment samples were obtained in this study. Microscopy was attempted, but no sporocysts or oocysts were detected in any of the samples. Of the 157 sediment samples, 19 samples tested positive via PCR amplification of the 18S rRNA gene. After cloning, sequencing and BLASTn analysis, two of the samples were found to have two different *Sarcocystis* species within the same sample, resulting in a total of 21 positive clones. Of the 20 clones obtained from river sediment, 13 were found to be *S. singaporensis*, three were *S. nesbitti,* three were *Sarcocystis* sp. YLL-2013 and one was an unidentified *Sarcocystis* species. One positive water tank sample contained *S. nesbitti* (Table 1).

**Table 1:** Summary of positive water samples. A total of 19 water samples were detected as positive. However, sample TIOW238 and TIOW307 contained a mixture of *Sarcocystis* spp. thus a total of 21 positive clones were obtained

Sample type	Village	No. of positive samples	Sample code (clone number)	*Sarcocystis* species	Potential definitive host	Potential intermediate host	Reference
Water tank	Salang	1	TIOW003 (5)	*S. nesbitti*	Reticulated python; Monocled cobra	*Macaca mulatta*; *Macaca fasicularis*	[5–7]
River	Salang	2	TIOW013 (3); TIOW020 (2)	*S. singaporensis*	Reticulated python	*Rattus* spp. and *Bandicota* spp.	[12]
		1	TIOW015 (7)	*S. nesbitti*	Reticulated python; Monocled cobra	*Macaca mulatta*; *Macaca fasicularis*	[5–7]
		1	TIOW022 (1)	*Sarcocystis sp.*	–	–	–
	Juara	10	TIOW026 (1); TIOW219 (2); TIOW220 (2); TIOW225 (1); TIOW228 (5); TIOW232 (3); TIOW235 (2); TIOW236 (5); TIOW243 (1); TIOW247 (10)	*S. singaporensis*	Reticulated python	*Rattus* spp. and *Bandicota* spp.	[12]
		1	TIOW238 (1)	*Sarcocystis* sp. 5 YLL-2013	Reticulated python	Undefined	[5]
		1	TIOW238 (10)	*Sarcocystis* sp. 1 YLL-2013	Reticulated python	Undefined	[5]
		1	TIOW249 (5)	*S. nesbitti*	Reticulated python; Monocled cobra	*Macaca mulatta*; *Macaca fasicularis*	[5–7]
	Ayer Batang	1	TIOW307 (3)	*Sarcocystis* sp. 5 YLL-2013	Reticulated python	Undefined	[5]
		1	TIOW307 (5)	S. *singaporensis*	Reticulated python	*Rattus* spp. and *Bandicota* spp.	[12]
		1	TIOW311 (10)	*S. nesbitti*	Reticulated python; Monocled cobra	*Macaca mulatta*; *Macaca fasicularis*	[5–7]

**Fig. 1:**
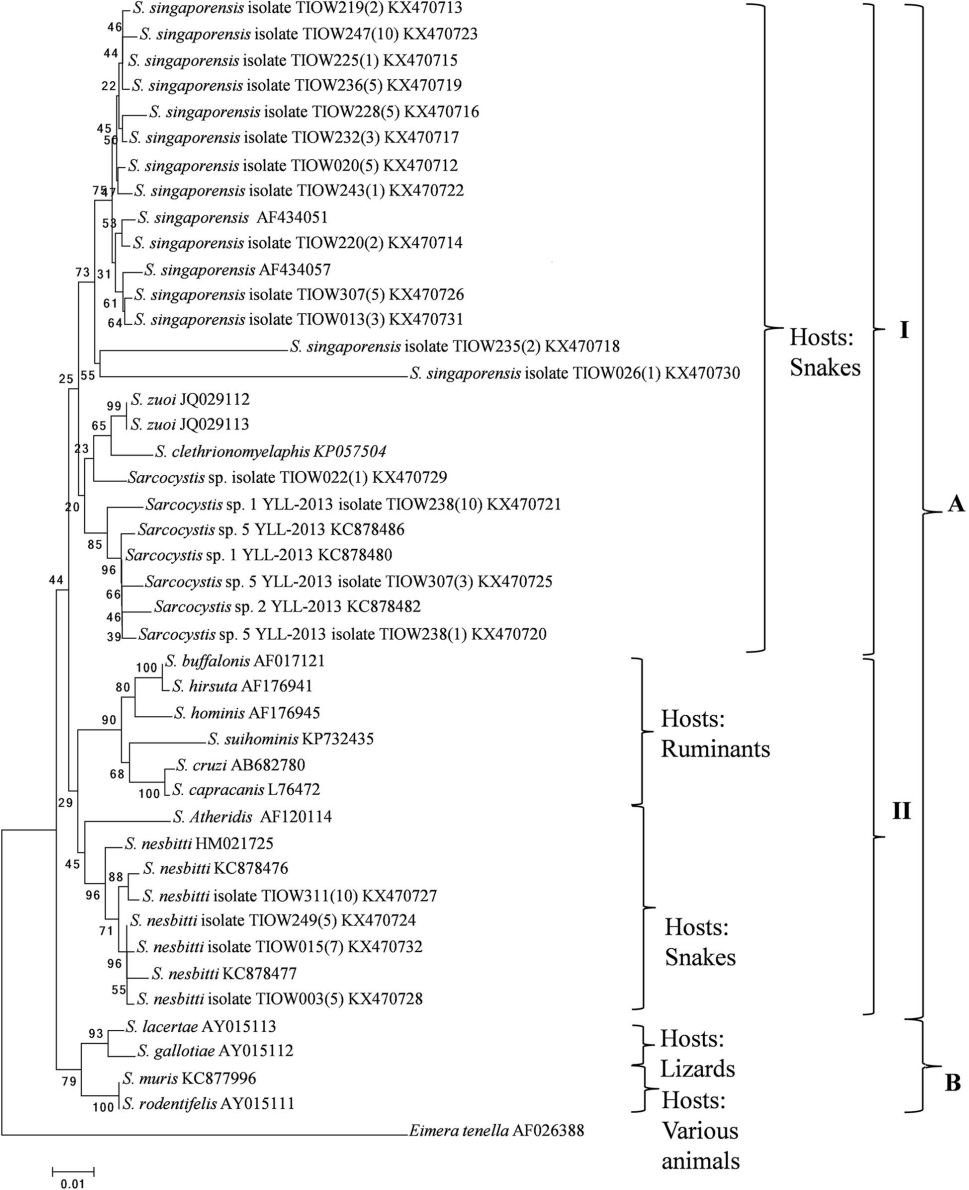
Neighbour-Joining tree constructed with MEGA6 using 18S rRNA gene sequences of *Sarcocystis* spp. Values represent percentage of replicate trees in which the associated sequences cluster together in the bootstrap test (1000 replicates)

Following species identification, the results were reappraised by comparing the 21 cloned sequences with another 31 sequences of the 18S rRNA gene from various other *Sarcocystis* species obtained from GenBank. This resulted in the construction of a Neighbour-Joining phylogenetic tree (Fig. 1) that was divided in to 2 clades (A and B). Clade A had two subclades, Subclade I and Subclade II, with Subclade I consisting of *Sarcocystis* species that infect snakes as definitive hosts, and Subclade II consisting of *Sarcocystis* species that infect snakes as definitive hosts and ruminants as intermediate hosts. Clade B consisted of *Sarcocystis* species that infect various hosts such as lizards and felines. All samples in this study, *S. singaporensis*, *S. nesbitti*, *Sarcocystis* sp. YLL-2013 and an unidentified *Sarcocystis* species, reside in Clade A.

## Discussion

This was a cross-sectional study aimed at providing evidence to support the hypothesis that *S. nesbitti* was transmitted via ingestion of contaminated water. Although screened water samples were found to be positive for *S. nesbitti*, a viability test was not conducted to ascertain that the sporocysts remained infective after being immersed in water. This was not done because microscopy failed to reveal any sporocysts. In future studies, however, if sporocysts are obtained, nucleic acid dyes could be used to stain sporocysts for a viability assay [10]. Furthermore, each sample was collected in a 50 ml Falcon tube, which is only a fractional representation of the entire water source from which it originated. Thus, this may give rise to false negatives and the results are likely an underestimation of the true prevalence of *Sarcocystis* species obtained from the various water sources. Although slightly unorthodox, this method was sensitive enough to detect *Sarcocystis* species in water samples where previous studies failed to do so when using standardised filtration methods [8]. Moreover, insufficient studies have been undertaken to elucidate the pros and cons of alternative water sampling methods in comparison to standardised methods [11]. This method was thus assumed to be adequate for the purpose of this study.

The majority of the water samples (14 out of 19 samples) was found to contain *S. singaporensis*. The life-cycle of *S. singaporensis* utilizes the reticulated python (*Python reticulatus*) as a definitive host and rodents from the genera *Rattus*, *Bandicota* and *Arvicanthis* as intermediate hosts [12]. Previous studies have shown that *S. singaporensis* is highly pathogenic to its intermediate host and thus could be used as a potential biocontrol agent [12]. Furthermore, *S. singaporensis* is the only snake-rodent *Sarcocystis* species for which an in vitro culture of the asexual stages has been established [13].

*Sarcocystis* sp. YLL-2013 was detected in three of the water samples. *Sarcocystis* sp. YLL-2013 was identified in 2013 when sporocysts were found in stool samples obtained from reticulated pythons [5]. Thus, the only known potential definitive host for *Sarcocystis* sp. YLL-2013 is the reticulated python, whereas intermediate host species have yet to be identified.

*Sarcocystis nesbitti* was found in four water samples; this species has previously been found in muscle tissue of rhesus monkeys (*Macaca mulatta*) and long-tailed macaques (*Macaca fascicularis*) [6, 7]. Of the two species, only *M. fascicularis* is found on Tioman Island [14]. The potential definitive host species for *S. nesbitti* are the monocled cobra (*Naja kaouthia*) and the reticulated python [5]. This was shown phylogenetically where snakes were suggested as intermediate hosts for *S. nesbitti* [15] and subsequently screening of snake faecal samples via PCR, revealed positive *S. nesbitti* fecal samples from a reticulated python and a monocle cobra [5]. However, caution should be taken as microscopy of the samples from that study failed to reveal any sporocysts [5] and no infection studies have yet to been undertaken. Thus, although the evidence is suggestive of the reticulated python and monocle cobra being the definitive host, this statement is still inconclusive. Of the potential definitive host species, only the reticulated python is found on Tioman Island [16, 17]. It is therefore hypothesised that on Tioman Island, *S. nesbitti* naturally cycles between reticulated pythons and long-tailed macaques, as definitive hosts and intermediate hosts, respectively. In future studies to identify the reservoir for *S. nesbitti* sporocysts on the island, reticulated pythons should be targeted as the main suspect. As snakes consume their prey whole, however, the selection criterion for such a study should include pythons of a certain size that are able to regularly consume monkeys, i.e. adult pythons. An alternative hypothesis proposed by Tappe holds that monitor lizards (*Varanus salvator*) could potentially be able to produce *S. nesbitti* sporocysts [18]. This was based on frequent sightings of these lizards at the villages where cases occurred and seasonal behavioural changes of these lizards that coincided roughly with the times of the outbreaks, among other reasons [18]. In this study, however, it was found that *S. nesbitti* does not cluster with other *Sarcocystis* species that utilise lizards as definitive hosts (Fig. 1). As *Sarcocystis* species in reptiles coevolved with their respective definitive hosts [19], *Sarcocystis* species are expected to cluster with other *Sarcocystis* species that share similar definitive hosts. Thus, Tappe’s hypothesis seems unlikely, but should not be discounted completely.

As *S. nesbitti* was found in a river sample and a water tank sample, this is the first finding to support the hypothesis that water was a potential medium for the transmission of *S. nesbitti* during the outbreaks. Prevention measures can now be taken by providing adequate treatment to water before consumption. It should be noted, however, that *Sarcocystis* sporocysts are chemically resistant to most disinfectants [1]. Fortunately, *Sarcocystis* sporocysts are susceptible to both high temperatures [10] and UV irradiation [20]. Thus, at the very least, water should be boiled before consumption and bottled water should be used for activities such as brushing teeth.

## Conclusion

This is the first study to provide evidence to support the hypothesis that the transmission of *S. nesbitti* during the 2011 and 2012 outbreaks was via ingestion of contaminated water. Three other *Sarcocystis* species were also detected in the water samples: *S. singaporensis*, *Sarcocystis* sp. YLL-2013 and an unidentified *Sarcocystis* species. This study has therefore identified potential areas in which preventive measures can be taken to prevent future outbreaks and provided information on other *Sarcocystis* species that inhabit Tioman Island.

## Abbreviations

AMS: Acute muscular sarcocystosis
BLAST: Basic Local Alignment Search Tool
DNA: Deoxyribonucleic acid
PCR: Polymerase chain reaction
